# Skipped wells and scientific error during fosfomycin agar dilution and broth microdilution lead to inconsistent minimal inhibitory concentrations and may be cause for reevaluating testing methods for *Klebsiella pneumoniae*

**DOI:** 10.1128/spectrum.04205-23

**Published:** 2024-06-28

**Authors:** Morgan L. Bixby, Ellora C. Daley, Lindsey B. Collins, Jenna M. Salay, Alexandra L. Bryson, Elizabeth B. Hirsch

**Affiliations:** 1 University of Minnesota College of Pharmacy, Minneapolis, Minnesota, USA; 2 Virginia Commonwealth University Health System, Richmond, Virginia, USA; City of Hope Department of Pathology, Duarte, California, USA

**Keywords:** *Klebsiella*, UTI, fosfomycin, broth microdilution, susceptibility

## Abstract

Despite the first-line recommendation of fosfomycin for uncomplicated urinary tract infections (UTIs), there are pressing barriers for optimizing its use for the treatment of non-*Escherichia coli* Enterobacterales UTI. There are no approved breakpoints for oral use against other Enterobacterales, and the recommended agar dilution (AD) reference method for minimal inhibitory concentration (MIC) determination is largely impractical. Using 160 clinical *Klebsiella pneumoniae* isolates, we sought to understand rates of skipped wells and MIC imprecision in broth microdilution (BMD) and how that compares to rates of error using AD. Though the Clinical and Laboratory Standards Institute refers to the skipped well phenomena in their recommendation against the use of BMD, there is a paucity of data on its frequency. While AD and BMD produced similar MIC_50/90_ values (32/256 µg/mL for AD and 64/256 µg/mL for BMD), essential agreement was poor. No-growth wells at concentrations below the MIC occurred in up to 10.9% of wells at a given concentration, as the most frequent scientific error. Growth in concentrations above the measured MIC occurred in up to 3.3% of wells and was seen within three dilutions of the MIC for BMD. Observation of single colonies either at or beyond the measured MIC for AD was also common and occurred up to 8.3% and 2.5% of the time, respectively. The frequent scientific error in both testing methods should prompt re-evaluation of AD guidelines and expansion of MIC testing methods for fosfomycin susceptibility testing, as poor agreement with another method prone to scientific error should not be the main detractor from BMD use.

**IMPORTANCE:**

Despite the recommendation of fosfomycin for uncomplicated urinary tract infections (UTIs), there are barriers for optimizing its use. There are no approved breakpoints for oral use against other Enterobacterales, and the recommended agar dilution (AD) reference method for MIC determination is largely impractical. The use of broth microdilution (BMD) for fosfomycin testing is not recommended by the Clinical and Laboratory Standards Institute due to unsatisfactory precision and skipped wells—occurrence of no-growth in a single well before the minimal inhibitory concentration (MIC)—and trailing endpoints. We sought to understand rates of skipped wells and growth at concentrations above measured MICs in BMD and how that compares to scientific error using AD. No-growth wells at concentrations below the MIC occurred in up to 10.9% of wells for BMD and single colonies at or beyond measured MICs for AD were also common. Frequent scientific error in both methods should prompt re-evaluation of both AD and BMD for fosfomycin susceptibility testing.

## INTRODUCTION

There is a current lack of oral antibiotics available to treat urinary tract infections (UTIs) caused by multidrug-resistant (MDR) pathogens. Fosfomycin—a first-line antibiotic for the treatment of uncomplicated UTI—is often used to treat UTI caused by non-*Escherichia coli* Enterobacterales (1–5) despite the absence of approved indications. The approval of fosfomycin in the United States includes only the oral treatment of *E. coli* and *Enterococcus faecalis*, but internationally the IV formulation is used broadly for all Enterobacterales (6). However, clinical outcomes data for oral treatment have demonstrated mixed results for treatment of non-*E*. *coli* MDR infections and these retrospective analyses are often difficult to interpret due to the absence of follow-up cultures or clinical outcomes (1–4, 7–11). These retrospective studies of patients with *K. pneumoniae* show that despite clinical microbiology testing showing *in vitro* susceptibility to fosfomycin—based on extrapolated breakpoints—treatment was successful less than half of the time (1, 4, 7, 8). These data highlight the discordance between the susceptibility testing results and clinical outcomes and whether current testing methods and interpretation protocols could be contributing factors.

Fosfomycin susceptibility testing recommendations are complex. Agar dilution (AD) is the reference method for both the Clinical and Laboratory Standards Institute (CLSI) and the European Committee on Antimicrobial Susceptibility Testing (EUCAST) (6, 12). Neither CLSI nor EUCAST supports the use of broth microdilution (BMD) for fosfomycin susceptibility testing (6, 13). The use of BMD for fosfomycin susceptibility testing is not recommended by CLSI due to unsatisfactory precision and skipped wells—occurrence of no-growth in a single well before the minimal inhibitory concentration (MIC)—and trailing endpoints (14, 15). However, the published data reporting on these phenomena are limited to fosfomycin studies where the only Enterobacterales included was *E. coli*, or to non-fosfomycin agents against other Enterobacterales (14, 16, 17).

AD is both time- and labor-intensive, and is not a feasible option for many clinical laboratories as a separate testing method for fosfomycin. It is more efficient for clinical microbiology laboratories to utilize BMD or automated systems which typically use a modified BMD method [Vitek 2 (bioMérieux) (18) and Phoenix (Becton Dickinson)] for MIC testing of clinical isolates when they are already in use for most other tested agents. These methods have been published in use globally; Brazil, Israel, Italy, South Africa, Turkey, and Poland utilize BMD/automated systems (19–24). Publications utilizing Vitek 2 and Phoenix for determining susceptibility have also extrapolated CLSI breakpoints despite the explicit recommendation against this practice of using BMD for fosfomycin susceptibility testing (12, 14). However, bioMérieux has successfully added fosfomycin to their Vitek 2 Gram-Negative Susceptibility Card following 510(k) U.S. Food and Drug Administration (FDA) clearance (18). The use of Vitek 2 in fosfomycin susceptibility testing has a listed indication for “antimicrobial susceptibility testing Gram-negative bacilli” but currently lists *E. coli* as the only Gram-negative organism for which the FDA recognizes fosfomycin activity (18). Finally, EUCAST has notably lower MIC breakpoints compared to CLSI’s oral fosfomycin breakpoints, with EUCAST lowering the susceptibility breakpoint for IV fosfomycin from 32 to 8 µg/mL in 2024 (Table 1).

**TABLE 1:** CLSI and EUCAST breakpoints for oral and intravenous (IV) fosfomycin formulations

	MIC (µg/mL)		
	Susceptible	Intermediate	Resistant
EUCAST^*a*^	≤8	NA	>8
CLSI^*b*^	≤64	128	≥256

^*a*^ Only for oral use of uncomplicated UTI caused by *E. coli* and intravenous use for *E. coli* infections originating in the urinary tract.

^*b*^ Only *E. coli* from the urinary tract and *E. faecalis*.

In light of the lack of published data for performance of BMD in fosfomycin susceptibility testing, we sought to study the rates of skipped wells and growth beyond the measured MIC observed in BMD and AD testing among a collection of 160 clinical *K. pneumoniae* isolates. We also sought to analyze the agreement of BMD to AD MIC.

## MATERIALS AND METHODS

### Bacterial isolates

This study included a convenience sample of 160 *K*. *pneumoniae* clinical isolates collected at four US locations from 2013 to 2016 (*n* = 80) (3, 25–27) and 2022 to 2023 (*n* = 80). Anatomical culture sites of the isolates included urine (*n* = 143), blood (*n* = 9), sputum (*n* = 5), and wounds (*n* = 3). Of these, 34 (21%) were confirmed to be ESBL-producing isolates (3, 25–27).

### Susceptibility testing and interpretation

**Fig 1:**
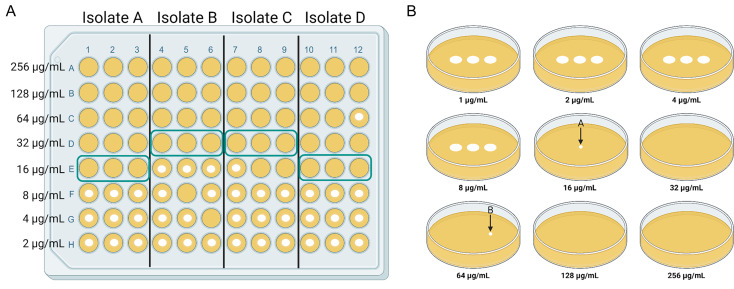
Example of the types of scientific error seen in broth microdilution testing (BMD) and agar dilution (AD). In panel A, the green box around three wells in the same row represents the measured MIC for a single biological replicate. Isolate A represents an isolate with no scientific error seen across the three columns. Isolate B represents an isolate with skipped wells at 8 µg/mL (F5) and 4 µg/mL (G6), where there is no-growth in a well that has growth in the concentration immediately above and below. Isolate C represents variable MIC where all three replicates do not share a MIC, but they are read at the lowest concentration where all three replicates have no-growth; thus, there is no-growth in two replicates at 16 µg/mL (E8 and E9). Isolate D represents single well growth above the measured MIC at 64 µg/mL (C12) when the MIC is read as the lowest concentration where all three wells have no-growth. In panel B, arrow A represents a single colony growth at the MIC (16 µg/mL) and arrow B (64 µg/mL) represents single colony growth at a concentration higher than the MIC.

Fosfomycin susceptibility was determined in biological duplicate and technical triplicate, on separate days, via AD and BMD (12, 27). A biological replicate was defined as a separate colony from the same clinical isolates used on a different day to create a saline suspension for both methods. A technical replicate was defined as use of the same diluted saline suspension for plating separate columns of wells for BMD or droplet for AD. The measured MIC was that of a single biological replicate, with each test read as the first concentration where there was no visible growth for all three technical replicates, and any wells with no growth (skipped wells or variable MIC) or growth at concentrations above that MIC were noted (Fig. 1). The final MIC used for analysis was the MIC shared by the two biological replicates or the higher of two MICs (if different) as long as the difference was within the acceptable error of ±1 dilution.

Test isolates were inoculated onto blood agar plates for overnight growth at 37°C. Single isolated colonies were used to inoculate sterile saline to a density of ~1.5 × 10^8^ CFU/mL as determined by optical density (OD_630_). For AD, the saline suspension was diluted to 2 × 10^6^ CFU in Mueller-Hinton II broth (MHB) (Fisher Scientific; Hampton, NH) where 5 µL (10^4^ CFU) of the MHB suspension was placed in triplicate onto a series of Mueller-Hinton agar (MHA) (Fisher Scientific; Hampton, NH) plates supplemented with fosfomycin (Fisher Scientific; Hampton, NH) plus 25 µg/mL of glucose-6-phosphate (G6P) (Neta Scientific Inc.; Hainesport, NJ). For BMD, the saline suspension was diluted to 10^6^ CFU/mL in MHB where 50 µL of the MHB suspension was placed into three columns of a 96-well plate with each row containing a different concentration of fosfomycin plus 25 µg/mL of G6P. The CFU/mL was confirmed by serial dilution and plating of the inoculum for each biological replicate. Test concentrations of fosfomycin ranged from 1 to 256 µg/mL for AD and 2 to 256 µg/mL for BMD (12, 27). *E. faecalis* ATCC 29212 (MIC: 32–128 mg/L) was used as a control strain, per Table 5A-1 of the CLSI M100, to capture the typical concentration range of our test isolates and was run in parallel with test isolates for each susceptibility test. Furthermore, to ensure appropriate G6P concentrations, *E. coli* ATCC 25922 was used to test each new batch of frozen G6P with fosfomycin using AD to confirm MIC with G6P potentiation. All tests were performed according to recommendations in the CLSI M100 document (12).

Due to the lack of established breakpoints for *K. pneumoniae*, breakpoints were extrapolated from CLSI and EUCAST fosfomycin breakpoints for *E. coli* (Table 1).

### Investigation of skipped wells and growth at concentrations above measured MIC

For BMD, the number and frequency of skipped wells, variable MIC, and growth at concentrations above the measured MIC were determined (Fig. 1A). As BMD was performed in triplicate (technical replicate), a no-growth well was defined as any well, or any two wells, with no visible growth at concentrations lower than the measured MIC. In a single replicate, a no-growth well with growth in concentrations above and below that well was considered a skipped well. Variable MIC was defined as one or more no-growth wells in concentrations immediately below the measured MIC. Growth at concentrations above the measured MIC during BMD testing was borrowed from the AD MIC definition, and defined as any visible growth at concentrations higher than the measured MIC, as these methods were both conducted in technical triplicate as opposed to CLSI’s recommendation of a single lane for BMD (15).

For AD, CLSI guidelines state that the MIC should be read at the first concentration where there is a single colony or no visible growth (Fig. 1B) (15). Thus, additional growth in AD was defined as a single colony at the measured MIC or countable colony growth at concentrations above the measured MIC.

The frequency of skipped wells, variable MIC, and growth above the measured MIC were analyzed by both final and measured MIC, since a one-dilution error was allowed in cases where the higher of the two dilutions was recorded as the final MIC. Thus, a final MIC may have been comprised of two different dilutions that were analyzed individually based on the measured MIC for each biological replicate.

### Correlation of susceptibility testing

Correlation between AD and BMD was calculated using AD as the reference method. Essential agreement (EA) was determined per CLSI guidelines and was achieved when the BMD MIC was within one dilution of the AD MIC (12, 15, 28).

## RESULTS

### Susceptibility testing and interpretation

The MIC ranges for AD and BMD were ≤1 to >256 µg/mL and ≤2 to >256 µg/mL, respectively (Table 2). While both methods resulted in the same MIC_90_ of 256 µg/mL, the MIC_50_ value was one dilution lower for AD as compared to BMD (32 µg/mL vs 64 µg/mL). The resulting interpretive categories of the isolates differed based on the method used. AD resulted in 8.8% (*n* = 14) by EUCAST and 80.6% (*n* = 129) by CLSI being considered susceptible. BMD resulted in 5.6% (*n* = 9) by EUCAST and 53.8% (*n* = 86) by CLSI being considered susceptible.

**TABLE 2:** Interpretation of fosfomycin susceptibility testing measurements

	Susceptible (S)	Intermediate (I)	Resistant (R)	MIC range (µg/mL)	MIC_50/90_ (µg/mL)
EUCAST breakpoints (*n* = 160)					
Agar dilution, *n* (%)	14 (8.8)	NA^*a*^	146 (91.3)	1 to >256	32/256
Broth microdilution, *n* (%)	9 (5.6)	NA	151 (94.4)	<2 to >256	64/256
CLSI breakpoints (*n* = 160)					
Agar dilution, *n* (%)	129 (80.6)	14 (8.8)	12 (7.5)	1 to >256	32/256
Broth microdilution, *n* (%)	86 (53.8)	32 (20.0)	42 (26.3)	<2 to >256	64/256

^*a*^ NA, not available from EUCAST.

### Investigation of skipped wells and growth at concentrations above measured MIC

**Fig 2:**
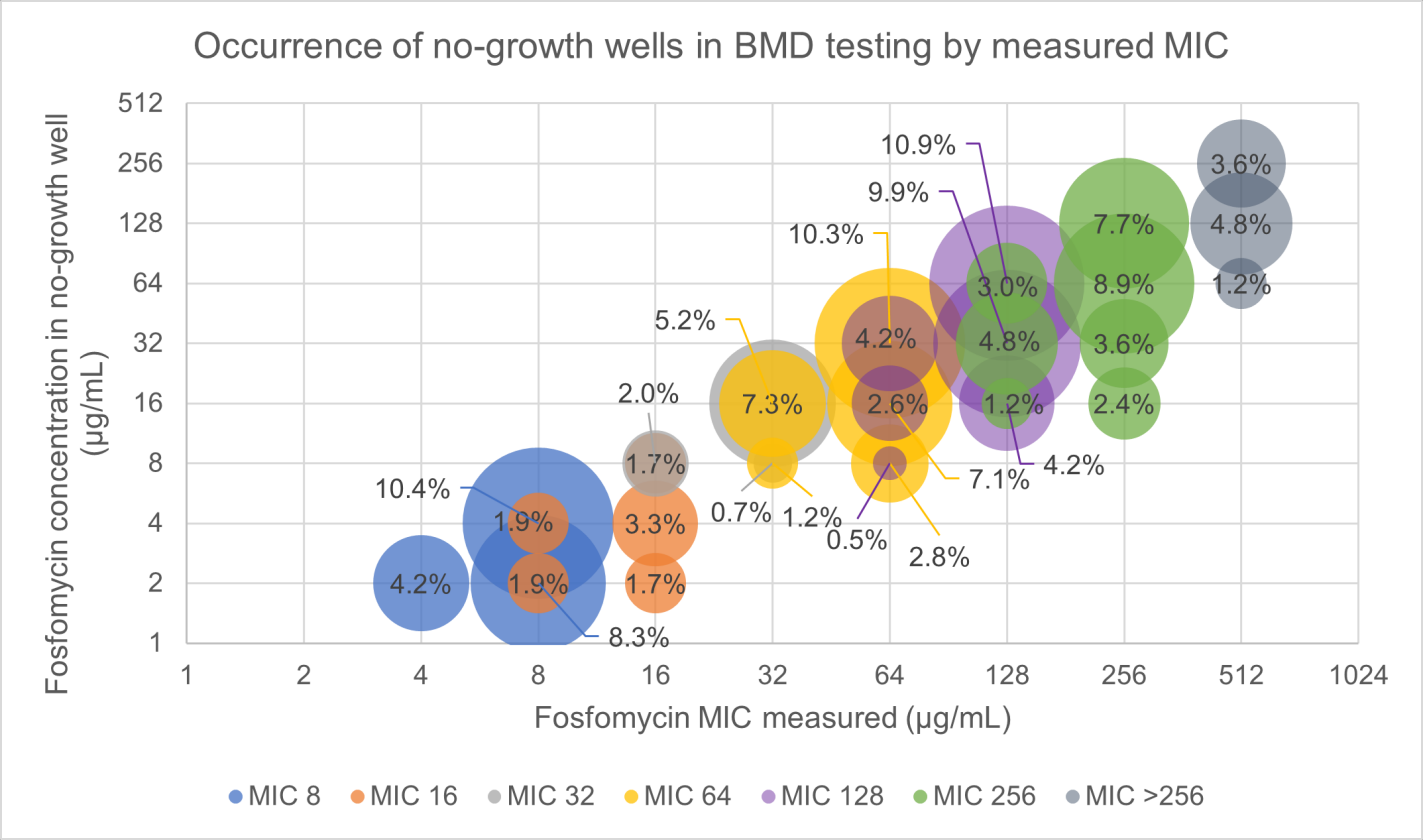
Occurrence of no-growth wells (skipped wells and MIC variability) in BMD testing by measured MIC. No-growth wells were analyzed in the frequency of the event by both final and measured MIC, as a one-dilution error was allowed where the higher of the two dilutions was recorded as the final MIC. Thus, a final MIC may have been comprised of two different dilutions that were analyzed individually based on the measured MIC for each biological replicate. The measured MIC is displayed by placement on the *x*-axis and final MIC is displayed by the data series color noted in the legend.

In BMD, imperfect susceptibility testing results in either skipped wells, MIC variability, or growth beyond the measured MIC occurred at all final MICs of 8 to >256 µg/mL. The most common of these types of scientific error was no-growth due to variable MIC during BMD testing, where up to 10.9% of wells at any given fosfomycin concentration had no-growth (Fig. 2). No-growth wells were most commonly seen in concentrations one dilution lower than the measured MIC. The most frequent examples of no-growth wells being those resulting from variable MIC: 10.3% of the wells at 32 µg/mL were skipped when the measured/final MIC was 64 µg/mL; 10.4% of wells at 4 µg/mL were skipped when the measured/final MIC was 8 µg/mL; and 10.9% of wells at 64 µg/mL were skipped when the measured/final MIC was 128 µg/mL.

**Fig 3:**
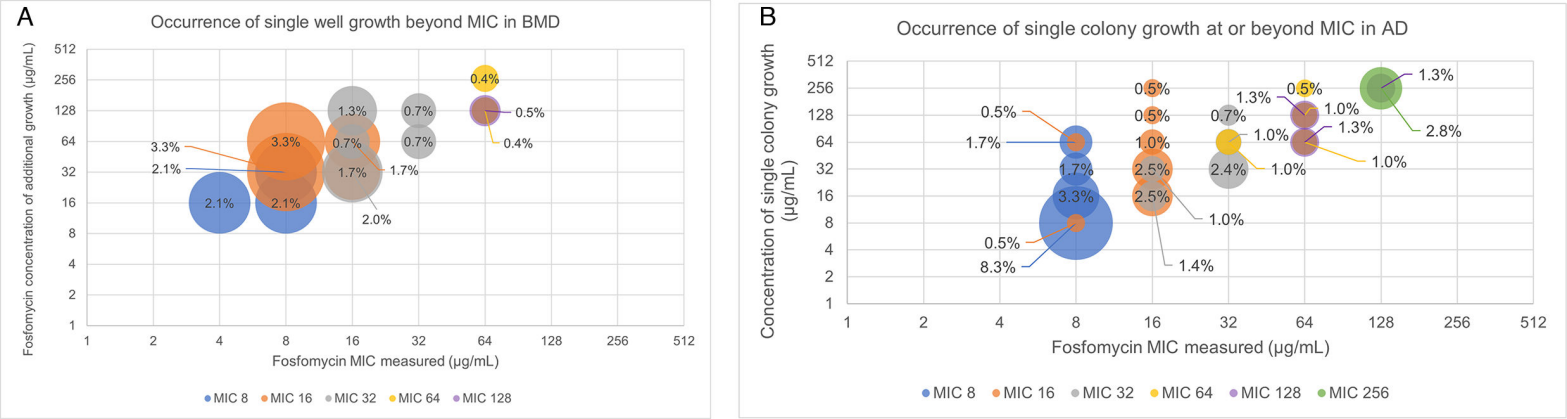
Occurrence of growth beyond the MIC measured among (**A**) BMD and (**B**) AD testing. Additional growth at concentrations higher than the MIC (single wells in BMD or single colonies in AD) were analyzed in the frequency of the event by both final and measured MIC, as a one-dilution error was allowed where the higher of the two dilutions was recorded as the final MIC. Thus, a final MIC may have been comprised of two different dilutions that were analyzed individually based on the measured MIC for each biological replicate. The measured MIC is displayed by placement on the *x*-axis and final MIC is displayed by the data series color noted in the legend.

The presence of growth in wells beyond the measured MIC was less common, with up to 3.3% of wells with concentrations beyond the measured MIC having growth (Fig. 3A). These wells beyond the MIC occurred at one to three dilutions higher than the final/measured MIC and were not seen at measured MICs ≥128 µg/mL.

During AD, the presence of single colony growth at or beyond the measured MIC occurred up to 8.3% of the time (Fig. 3B). The presence of single colony growth was highest at the measured MIC and occurred between 0.5% and 3.3% of the time beyond the measured MIC. Single colony growth beyond the MIC was observed at nearly all fosfomycin concentrations (higher than the MIC) and was not limited by the measured MIC of the isolate. None of these phenomena were observed across the numerous replicates of the QC strain, *E. faecalis* ATCC 29212.

### Correlation of susceptibility testing

**Fig 4:**
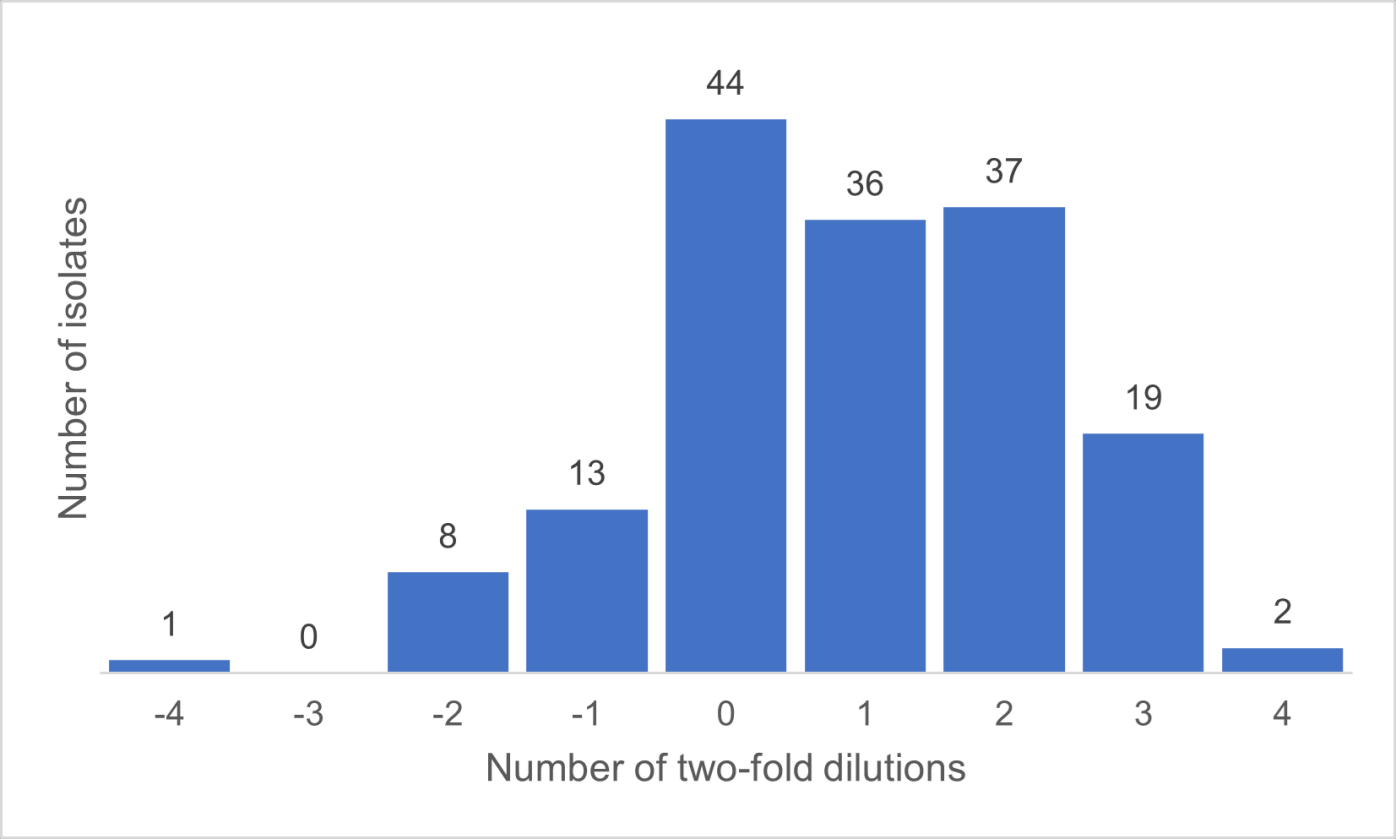
Discrepancy in essential agreement using BMD with AD as the reference method.

The two methods had 58.1% (*n*/*N* = 93/160) EA for this collection when using AD as the reference method. Of isolates that did not have EA, 92.1% (*n*/*N* = 58/67) resulted from BMD having a higher MIC than AD (Fig. 4).

## DISCUSSION

Available published data used to justify BMD as an unsuitable fosfomycin susceptibility testing method due to skipped wells is limited to a single study not including any non-*E. coli* Enterobacterales (14). While some internal CLSI data dating back to the original fosfomycin breakpoints review exist, these data are not widely available to help inform *K. pneumoniae* breakpoints should the development of IV fosfomycin continue forward in the United States (29). With the recent FDA clearance of fosfomycin being added to the Vitek 2 (bioMérieux) Gram-negative panel, it is important to study the implications of its extrapolated use for non-*E. coli* Enterobacterales among a set of clinical isolates (18). In addition, the only universally approved MIC testing method, AD, is time-intensive and not feasible for most clinical laboratory settings.

This study sought to compare the rates of skipped wells and growth beyond the measured MIC in a collection of clinical *K. pneumoniae* isolates (*n* = 160) for both BMD and AD fosfomycin susceptibility testing. The susceptibility testing resulted in different interpretations based on the extrapolated EUCAST and CLSI breakpoints with 8.8%–80.6% considered susceptible by AD and 5.6%–53.8% considered susceptible by BMD. Both testing methods resulted in MIC measurements that spanned the upper and lower limits of their tested concentrations, from 1 to >256 µg/mL and ≤2 to >256 µg/mL for AD and BMD, respectively.

Despite the different interpretations, no-growth wells in BMD were observed up to 10.9% of the time with a greater likelihood of the no-growth wells occurring closer to the MIC measured. BMD growth beyond the measured MIC was observed less frequently at up to 3.3% of the time, and only occurred within three dilutions of the MIC measured. During AD testing, single colonies at the MIC occurred up to 8.3% of the time, with single colonies beyond the MIC being observed up to 3.3% of the time. While there is not a comparable phenomenon for skipped wells in AD, the rates of growth after MIC for both methods were similar.

The MIC measured using BMD was often higher than those measured by AD for the same isolate. In contrast, the *E. coli* isolates (*n* = 365) used by bioMérieux for the validation of the Vitek 2 card had categorical agreement rates of ≥98.8% with no major or very major error, which were well within the acceptable error rates (18).

Fuchs et al. studied the precision of AD and BMD utilizing ATCC control strains to determine how frequently a MIC was recorded for that strain per the two methods (14). They found that for *E. coli* ATCC 25922, 95% of the observed MIC fell between the measurements of 0.5–2 µg/mL for AD and 0.5–8 µg/mL for BMD, thus showing BMD had trailing endpoints. This study also referenced that “frequent skipped wells” contributed to the lack of precision when using the BMD method but did not further elaborate. The authors attributed the lack of precision with BMD to variability in inoculum between 1.6 × 10^5^ and 1.2 × 10^6^ CFU/mL but did not explain the methods used to predict the inoculum concentration used to reduce variability, whereas our study used optical density and colony counts to ensure that all concentrations plated were close to 10^6^ CFU/mL per CLSI guidelines.

Abbott et al. saw a similar pattern to the current study where the MIC was often several dilutions higher when using BMD as compared to AD for 20 *K. pneumoniae* isolates (30). All the isolates in their study had a *fosA*-positive genotype, many of which screened positive for fosfomycin heteroresistance not attributable to *fosA* (30). They also demonstrated no upregulation of *fosA* expression after exposure to fosfomycin as compared to baseline. In another study by Abbott et al., heteroresistance was frequently found among the 20 wild-type *K. pneumoniae* isolates (defined as less than the upper limit of wild-type distribution; 64 µg/mL) (31). Using an *in vitro* bladder infection model simulating a single 3 g dose of fosfomycin, isolates with a MIC >2 µg/mL had a probability of target attainment <90% using Monte Carlo simulation, indicating this regimen likely lacks clinical efficacy for *K. pneumoniae* UTI (31). The presence of these scientific errors and higher MIC may be indicative of heteroresistance, like that seen by Abbott et al., which is better captured by BMD than AD, due to the higher quantity of CFU plated using BMD, thus better estimating the MIC of the heteroresistant population as a whole.

Strengths of this study include the large collection (*n* = 160) of phenotypically diverse isolates from multiple geographic locations, and the detailed analysis of growth beyond the measured MIC for both BMD and AD in addition to skipped wells and variable MIC. This is an area of fosfomycin susceptibility testing with few publications, and a greater understanding of the unreliability of BMD is needed should the use of automated methods such as Vitek 2 and Phoenix continue to be used for testing. Furthermore, the use of three technical replicates among BMD made the methodology more in line with reference method guidelines; it also introduced more opportunities to capture the scientific error than the CLSI standard one column (no technical replicates), offering both limitations and strengths to the analysis. While the current study takes a deeper look into the inaccuracy of fosfomycin susceptibility testing against *K. pneumoniae,* the ability to make greater assessments and conclusions on the skipped well phenomenon in fosfomycin susceptibility testing is limited due to a lack of inclusion of other Enterobacterales species.

Both BMD and AD resulted in consistent scientific error when reading the results of each method for this collection, though BMD resulted in more frequent reliability problems via skipped wells, variable MIC, and growth at concentrations above the MIC when compared to growth at or beyond the measured MIC in AD. With antibiotic susceptibility testing needing to move toward more rapid and less laborious methods to combat antibiotic resistance, poor agreement between the BMD method with a reference method having similar rates of scientific error affecting the final MIC should not be the major reason to preclude its use. Instead, the results of this study suggest that re-evaluation of guidelines associated with AD as the reference method, and expansion of approved MIC testing methods available for fosfomycin are needed.
